# Green propolis extract attenuates acute kidney injury and lung injury in a rat model of sepsis

**DOI:** 10.1038/s41598-021-85124-6

**Published:** 2021-03-15

**Authors:** Marcelo Augusto Duarte Silveira, José Manuel Condor Capcha, Talita Rojas Sanches, Roberto de Sousa Moreira, Margot S. Garnica, Maria Heloisa Shimizu, Andresa Berretta, Flávio Teles, Irene L. Noronha, Lúcia Andrade

**Affiliations:** 1 Laboratory of Basic Science in Renal Diseases, Division of Nephrology, University of São Paulo School of Medicine, São Paulo, Brazil; 2 University of Goiás, Catalão, Brazil; 3 Laboratory of Cellular, Genetic, and Molecular Nephrology, Division of Nephrology, University of São Paulo School of Medicine, São Paulo, Brazil; 4 Laboratório de Pesquisa, Desenvolvimento e Inovação (P, D & I), Apis Flora Industrial e Comercial Ltda, Ribeirão Preto, Brazil; 5 School of Medicine, Federal University of Alagoas, Maceió, Brazil

**Keywords:** Kidney, Infection

## Abstract

Sepsis is the leading cause of acute kidney injury (AKI) and lung injury worldwide. Despite therapeutic advances, sepsis continues to be associated with high mortality. Because Brazilian green propolis (GP) has promising anti-inflammatory, antioxidant, and immunomodulatory properties, we hypothesized that it would protect kidneys and lungs in rats induced to sepsis by cecal ligation and puncture (CLP). Male Wistar rats were divided into groups—control (sham-operated); CLP (CLP only); and CLP + GP (CLP and treatment with GP at 6 h thereafter)—all receiving volume expansion and antibiotic therapy at 6 h after the procedures. By 24 h after the procedures, treatment with GP improved survival, attenuated sepsis-induced AKI, and restored renal tubular function. Whole-blood levels of reduced glutathione were higher in the CLP + GP group. Sepsis upregulated the Toll-like receptor 4/nuclear factor-kappa B axis in lung and renal tissues, as well as increasing inflammatory cytokine levels and macrophage infiltration; all of those effects were attenuated by GP. Treatment with GP decreased the numbers of terminal deoxynucleotidyl transferase-mediated deoxyuridine triphosphate nick-end labeling-positive cells in renal and lung tissue, as well as protecting the morphology of the renal mitochondria. Our data open the prospect for clinical trials of the use of GP in sepsis.

## Introduction

Sepsis results in high costs for the health care system^1^. Acute kidney injury (AKI) occurs in 20–40% of patients with sepsis treated in the intensive care unit and is often present in those with multiple organ dysfunction^2–5^, among whom mortality can be as high as 60%^4^. The main measures for the prevention of AKI are the early use of antibiotics, together with hydration vasoactive drug administration. Increased knowledge of the pathophysiology of sepsis-induced AKI, which involves inflammation, oxidative stress, tubular cell damage, dysregulated microcirculation, morphofunctional alterations in the mitochondria, and apoptosis, has made it possible to devise new interventions^6–9^. Lung dysfunction is also a common challenge in patients with sepsis, potentially leading to acute lung injury and acute respiratory distress syndrome (ARDS). Although new evidence suggests that the mortality associated with acute lung injury/ARDS has declined recently, it continues to be approximately 26% in the United States^10^. In mice, the use of the cecal ligation and puncture (CLP) model results in intensive secretion of cytokines, altered respiratory function, pulmonary edema, and accumulation of inflammatory cells^11^.

Toll-like receptors (TLRs), innate immune system effectors that are present not only in defense cells but also in renal proximal tubular cells, recognize damage-associated and pathogen-associated molecular patterns^5,8^. Stimuli such as oxidative stress and TLR4 signaling activate nuclear factor-kappa B (NF-κB), thus triggering cytokine transcription and release, which result in inflammation^5,7,12^. There is evidence that inhibition of the TLR4/MyD88 signaling pathway ameliorates sepsis-associated ARDS in rats by regulating macrophage activation^13^.

Mitochondria, which generate metabolic energy through adenosine triphosphate production, undergo morphofunctional changes during sepsis^8,9^. The mitochondrial dysfunction occurring in sepsis is not yet fully understood, nor are the mechanisms of action of the latest drugs that protect against sepsis^14^. In sepsis, expression of endothelium-derived nitric oxide synthase (eNOS) is reduced, resulting in microcirculatory changes^5,7,9^.

Propolis is a natural resin produced by bees as a protection against macroscopic and microscopic invaders. It contains a number of substances with medicinal properties, having long been used by various populations^15,16^. In Brazil, there are 13 different types of propolis, which contain substances with great potential for the treatment of various diseases^16^. Green propolis, found mainly in southeastern Brazil, contains caffeic acid phenethyl ester (CAPE), *p*-coumaric acid, trans-Cinnamic acid, artepillin C, aromadendrin, and quercetin, all of which have documented antioxidant, anti-inflammatory, immunomodulatory, anticancer, and antimicrobial properties^15–21^.

In a rat model of AKI, red propolis was found to be renoprotective, reducing oxidative stress and preserving the endothelium^17^. Green propolis has been shown to reduce proteinuria and urinary monocyte chemoattractant protein-1 in patients with diabetic or nondiabetic chronic kidney disease^22^. Other authors have described the immunomodulatory properties of Brazilian propolis^16^. However, to our knowledge, there have been no studies evaluating its role in the attenuation of sepsis-induced AKI.

The objective of the present study was to determine whether green propolis protects against renal and pulmonary damage in the CLP model of sepsis. We hypothesized that treatment with green propolis would reduce oxidative stress, modulate inflammation, and preserve endothelial function.

## Methods

### Ethical aspects

The study was approved by the University of São Paulo School of Medicine Ethics Committee on the Use of Animals (Reference no. 115/15). All procedures were performed in strict compliance with local institutional guidelines and with well-established national standards for the handling and care of laboratory animals (Normative Resolutions of the Brazilian National Council for the Monitoring of Animal Experimentation), as well as with the Guide for the Care and Use of Laboratory Animals published by the US National Institute of Health (NIH Publication No. 85-23, 2011 revision). The study design and the presentation of the data are in accordance with the Animal Research: Reporting of In Vivo Experiments guidelines (https://arriveguidelines.org/).

### Characterization of the propolis

The green propolis extract employed was produced and kindly provided by the Apis Flora Company (Ribeirão Preto, Brazil; Patent no. PI 0405483-0, published in Revista de Propriedade Industrial n. 1778, Jan 2, 2005). The ethanol extract of propolis employed (EPP-AF) has a shelf life of four and a half years under stable conditions.

**Figure 1:**
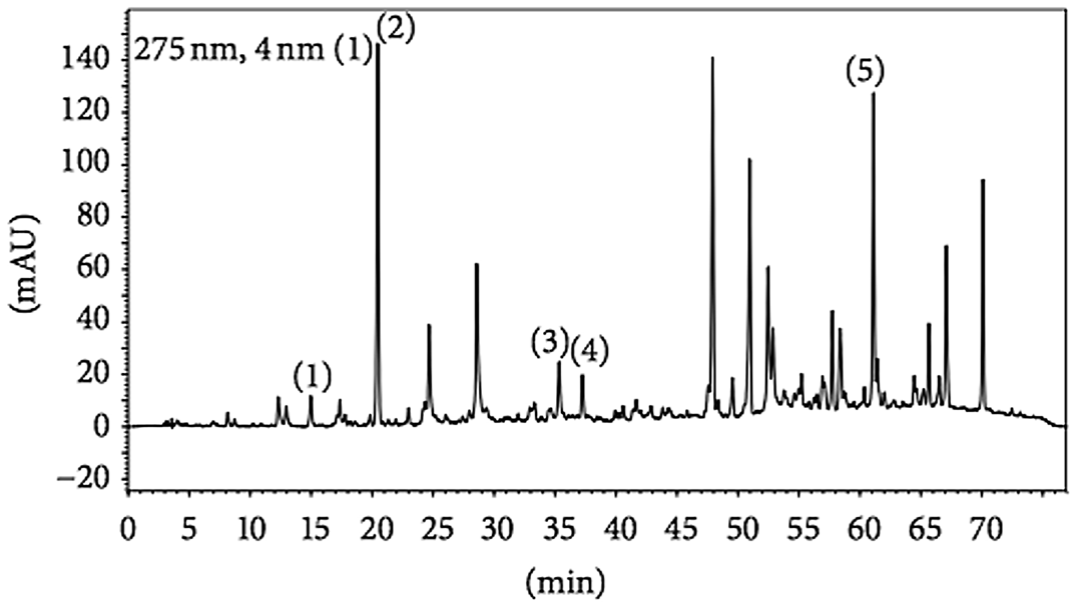
Fingerprint analysis of the ethanol extract of propolis. Chromatograms were plotted at 275 nm with reverse-phase high-performance liquid chromatography on a C18 column (4.6 mm × 250 mm; particle diameter, 5 µm; pore diameter, 100 Å) and gradient elution with methanol and acidic water (pH 2.7). The chromatographic profile includes the following compounds: (1) caffeic acid phenethyl ester (in approximately 15 min); (2) *p*-coumaric acid (in approximately 20 min); (3) trans-Cinnamic acid (in 35–36 min); (4) aromadendrin (in 38 min); and (5) artepillin C (in 61–62 min).

The propolis extract was analyzed in a high-performance liquid chromatography system (i-Series Plus; Shimadzu, Kyoto, Japan) equipped with a system controller, a quaternary pump, and a diode-array detector. The data were analyzed with LC solution software, version 1.21 SP1 (Shimadzu). As can be seen in Fig. 1, we used a 4.6 mm × 250 mm column with a particle diameter of 5 µm and a pore diameter of 100 Å (Shim-Pack CLC-ODS; Shimadzu).

### Animals and experimental protocol

Male Wistar rats, 8 weeks of age with a body weight (BW) of 250–300 g, were obtained from the animal facilities of the University of São Paulo School of Medicine. The animals were housed in cages with ad libitum access to water and food. The rats were randomly divided into three groups: control, comprising sham-operated rats; CLP, comprising rats submitted to CLP only; and CLP + GP, comprising rats submitted to CLP and receiving green propolis extract in the form of a powder (500 mg/kg BW, diluted in 5 ml of saline)^23,24^, administered by intraperitoneal (I.P.) injection at 6 h after the procedure. Rats in the control group received 5 ml of saline solution, by I.P. injection, at 6 h after the sham procedure. All experiments were conducted at 24 h after the procedures.

We conducted our study in three phases. In the first phase, we conducted a survival study using ten rats per group. In the second study, we evaluated the glomerular filtration rate (GFR, determined by quantifying inulin clearance), using seven control group rats, eight CLP group rats, and eight CLP + GP group rats. In the third phase, we performed metabolic cage studies, analyzing the blood and urine samples collected, together with histological analysis, immunohistochemistry, and Western blotting, using eight rats per group.

### CLP

As previously described^8,9^, we anesthetized the rats by injecting xylazine (7 mg/kg BW, I.P.) and ketamine (70 mg/kg BW, I.P.), after which we made a midline incision to expose the cecum and placed a 4–0 silk ligature at 1.5 cm from the cecal tip. A 16-gauge needle was used in order to make two punctures in the cecum, which was then squeezed gently to ensure that there was leakage of the cecal contents. We closed the abdominal incision, in two layers, using 3–0 nylon sutures. Control group rats underwent a sham procedure (without ligation or puncture). To ensure adequate fluid resuscitation, we injected each rat with saline solution 0.9% (25 ml/kg, I.P.) immediately after the procedure. At 6 h after the procedure, we administered an additional 25 ml/kg (I.P.) of saline solution 0.9% and 14 mg/kg (I.P.) of imipenem/cilastatin (Merck Sharp & Dohme, West Point, PA, USA) as antibiotic therapy.

### Inulin clearance

We measured inulin clearance at post-procedure hour 24. As previously described^7,25,26^, we injected each rat with thiopental sodium (50 mg/kg BW, I.P.) to induce anesthesia. Tracheas were cannulated, during spontaneous breathing, with a PE-240 catheter. We inserted a PE-60 catheter into the right carotid artery to collect blood samples. We then made an incision in the suprapubic region and cannulated the urinary bladder with a PE-240 catheter to collect urine samples. Through the jugular vein, we administered a loading dose of inulin (100 mg/kg BW) at the end of the surgical procedure, thereafter infusing inulin (10 mg/kg BW) at a constant rate of 0.04 ml/min. At 30-min intervals, we collected urine samples (n = 3). Before and after the experiment, we collected blood samples (0.3 ml and 4–6 ml, respectively). We employed the anthrone method to quantify inulin in urine and in plasma.

### Biochemical analysis

We performed gravimetry to determine the volume of each 24-h urine sample. To remove suspended material, we centrifuged the urine samples in aliquots, after which we analyzed the supernatants. A freezing-point osmometer (3D3; Advanced Instruments, Norwood, MA, USA) was used in order to determine urine osmolality. Flame photometry was used in order to determine the levels of sodium and potassium in serum and urine, whereas kinetic methods were used in order to determine serum levels of urea, as well as serum and urinary levels of creatinine.

### Urinary neutrophil gelatinase-associated lipocalin

Urinary neutrophil gelatinase-associated lipocalin (NGAL) was measured by using a commercially available enzyme-linked immunosorbent assay kit (046; BioPorto Diagnostics, Gentofte, Denmark).

### Reduced glutathione in blood

To determine reduced glutathione (GSH) content in whole blood, we used the Sedlak and Lindsay method^27^. In brief, four volumes of ice-cold 5% (W/V) metaphosphoric acid were added to each whole blood sample, after which we centrifuged the sample for 3 min at 14,000*g*. We then added Ellman’s reagent to the supernatant and measured the spectroscopic absorbance of the yellow pigment at 412 nm. Using a cysteine standard curve, we determined the whole-blood GSH level, which is expressed in micromoles per milliliter.

### Isolation of total protein

To isolate total protein in kidney samples, we used a homogenizer (PT 10/35; Metrohm, Herisau, Switzerland) and an ice-cold isolation solution containing 200 mM mannitol, 80 mM HEPES, and 41 mM potassium hydroxide (pH 7.5), together with a protease inhibitor cocktail (Sigma-Aldrich, St. Louis, MO, USA). We centrifuged the homogenates at 2000*g* and 4 °C for 15 min to remove nuclei and cell debris. We determined protein concentrations by using a Bradford assay (Bio-Rad Protein Assay Kit; Bio-Rad Laboratories, Hercules, CA, USA).

### Immunoblotting

To perform immunoblotting, we ran kidney samples on polyacrylamide mini-gels. Using electroelution, we transferred the proteins to polyvinylidene difluoride membranes (GE Healthcare, Little Chalfont, UK), after which we blocked the blots with 5% nonfat dry milk in Tris-buffered saline. We then incubated the blots overnight with antibodies against the following: β-actin (1:200,000; Abcam, Cambridge, MA, USA); manganese superoxide dismutase (MnSOD, 1:10,000; Cayman Chemical, Ann Arbor, MI, USA); the Na-K-2Cl cotransporter (NKCC2) and TLR4 (1:500 and 1:1000, respectively; Santa Cruz Biotechnology, Dallas, TX, USA); and NF-κB (1:500; Abcam). To visualize the labeling, we used a horseradish peroxidase-conjugated secondary immunoglobulin G (IgG) antibody—anti-goat (1:10,000), anti-mouse (1:2000), or anti-rabbit (1:2000)—obtained from Sigma-Aldrich, together with enhanced chemiluminescence detection (GE Healthcare) and the Alliance 4.2 imaging system (UVItec, Cambridge, UK). Quantitative analysis of the antibodies was performed with densitometry, the bands being normalized to β-actin expression.

### Evaluation of cytokines in renal tissue

We used a multiplex cytokine assay kit (Bio-Plex Rat 9-Plex; Bio-Rad, Hercules, CA, USA) to determine the levels of interferon gamma (IFN-γ), interleukin (IL)-1β, IL-12p70, (IL)-17A, and tumor necrosis factor (TNF)-α in renal tissue. We read the assay on the Bio-Plex suspension array system and analyzed the data with Bio-Plex Manager software, version 4.0 (Bio-Rad). We standardized the cytokine values by the ratio of the amount of protein in renal tissue, and the results are expressed in picograms per milligram.

### Evaluation of macrophage infiltrate in renal tissue

As previously described^7^, samples were set in paraffin blocks and cut into 4-mm sections for macrophage infiltrate (CD68) immunostaining. The sections were deparaffinized, after which endogenous peroxidase activity was blocked with 0.3% H_2_O_2_ in water at room temperature for 10 min. The sections were then incubated with a CD68 antibody (ED1 clone, 1:100; AbD Serotec, Oxford, UK) overnight at 4 °C, after which they were incubated with biotinylated mouse anti-rat IgG at room temperature for 30 min. To detect the reaction product, we used avidin-biotin-peroxidase complex (Vector Laboratories, Burlingame, CA, USA). After developing the color reaction, with 3,3-diaminobenzidine (Sigma-Aldrich), we counterstained the sections with methyl green.

We analyzed 30 grid fields (0.087 mm^2^ each) per kidney cortex, counting the number of infiltrating CD68 + cells in each field, on the basis of which we calculated the mean numbers in the renal cortical tubulointerstitium and the mean counts per kidney. We stained 4-µm histological sections of renal tissue with hematoxylin-eosin or Masson’s trichrome, in order to examine them under light microscopy. The images obtained by microscopy were captured on video via an image analyzer (Axiovision; Carl Zeiss, Eching, Germany), in order to perform the histomorphometric analysis.

### Immunohistochemistry

Cell death in renal tissue was evaluated through the use of immunohistochemistry to identify terminal deoxynucleotidyl transferase-mediated deoxyuridine triphosphate nick-end labeling (TUNEL), with an in situ cell death detection kit (POD; Roche, Penzberg, Germany). We quantified TUNEL + cells in 30 randomly selected fields.

### Mitochondrial morphology in renal tissue

To identify ultrastructural alterations in mitochondria, we performed electron microscopy (at a magnification of approximately 5000 ×) using one of two electron microscopes—a JEM 1010 (JEOL, Tokyo, Japan) or a TECNAI-10 (Philips, Eindhoven, The Netherlands). At 24 h after the procedures (CLP or sham surgery), renal cortex tissue samples were collected (from three rats per group) and fixed in 3% glutaraldehyde. Purified mitochondrial preparations were obtained, pelleted, and fixed in 2.5% glutaraldehyde in phosphate-buffered saline (PBS, pH 7.4) at 4 °C. The specimens were postfixed with 2% osmium tetroxide in 0.1 M phosphate buffer (pH 7.4), dehydrated, embedded in epoxy resin, and cut into thin (90-nm) sections.

### Leukocyte transmigration in the lungs

As previously described^28^, for indirect quantification of the recruitment or transmigration/activity of neutrophils in the lungs, we measured the enzyme myeloperoxidase activity, expressed in arbitrary units per gram. We homogenized lung tissue samples in PBS (50 mM, pH 6.0) containing hexadecyltrimethylammonium bromide (0.5%, W/V), after which we centrifuged the resulting homogenate for 20 min (at 10,000*g* and 4 °C) and used the supernatant in the enzyme activity assay. The enzymatic reaction was performed for 5 min in the presence of o-dianisidine (1.6 mM) and H_2_O_2_ 0.1% (in 80 mM phosphate buffer, pH 5.4). Absorbance at 450 nm is presented as optical density/tissue weight in grams.

### Immunofluorescence evaluation of apoptosis and TLR4 expression in lung macrophages

A cell death detection kit with TUNEL (Roche) was used in order to evaluate apoptosis in lung cells. For the expression of the CD68 and TLR4 in lung tissue, the primary antibodies were mouse anti-CD68 (ED1 clone, cat no. MCA341R; AbD Serotec) and rabbit anti-TLR4 (polyclonal, cat. no. Ab13556; Abcam), respectively. A double-labeling technique was used for CD68 and TLR4. The primary antibodies were added at a 1:50 dilution in PBS and incubated overnight at 4 °C. The samples were then washed three times in PBS for 10 min each, after which they were incubated in the secondary antibodies—Alexa 488 and Alexa 546 for CD68 and TLR4, respectively (1:200 for both; Molecular Probes, Eugene, OR)—for 1 h at room temperature. The slides were then washed three times in PBS and mounted with DAPI (10 µg/ml in 1:1 glycerol PBS, cat. no. D1304; Invitrogen, Carlsbad, CA, USA).

The slides were stored in darkness and chilled until they were photographed, usually on the same day. Each image was scanned with a fluorescence microscope (Eclipse 80i; Nikon, Tokyo, Japan) in 10 fields, with 380 nm (DAPI), 488 nm, and 546 nm filters, at a magnification of 400 × .

### Statistical analysis

Differences among the means of multiple parameters were analyzed by one-way ANOVA, followed by the Student–Newman–Keuls test. Quantitative data are expressed as mean ± SEM, and values of *p* < 0.05 were considered statistically significant. Survival analyses were compared by a log-rank test. The statistical software used was GraphPad Prism, version 6 (GraphPad Inc., La Jolla, CA, USA).

## Results

### Effects of green propolis on survival

**Figure 2:**
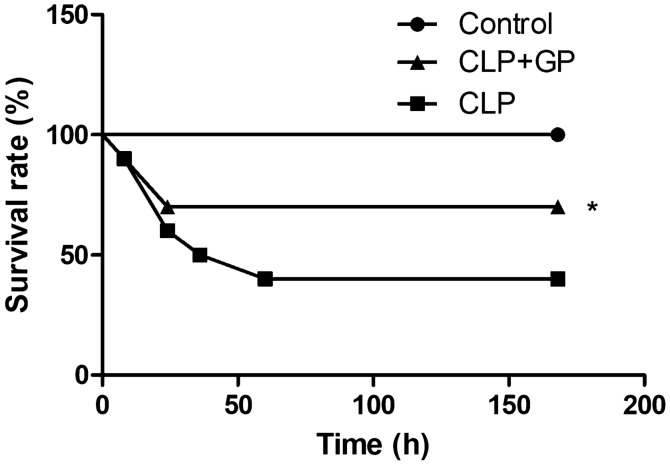
Effects of propolis on survival. *p < 0.05 versus CLP.

Mortality was significantly lower in the CLP + GP group than in the CLP group. The 7-day survival rate was 70% in the CLP + GP group and 40% in the CLP group (*p* < 0.05; Fig. 2).

### Effects of green propolis on sepsis-induced AKI

**Figure 3:**
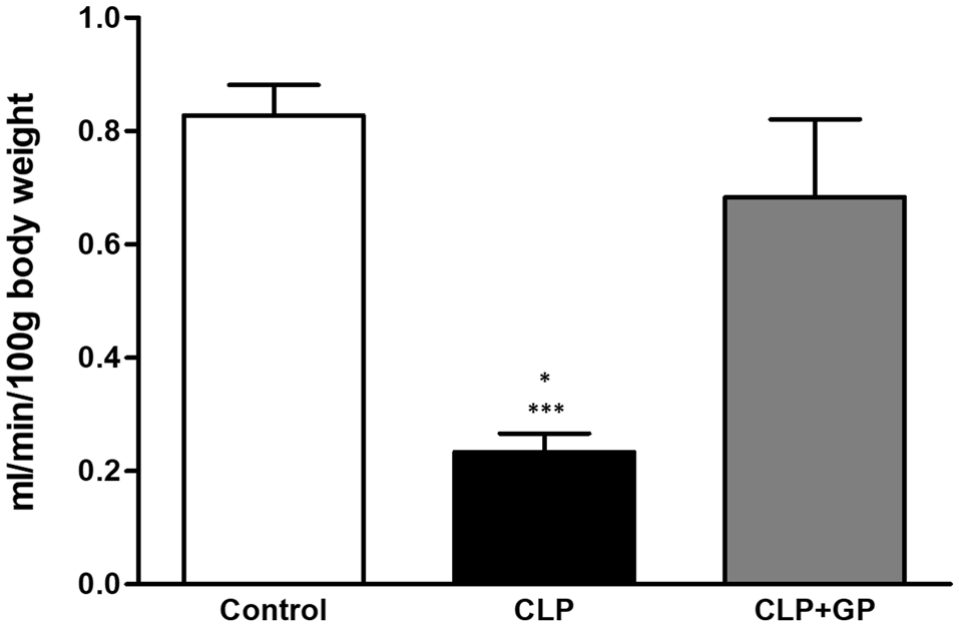
Renal function measured by inulin clearance (glomerular filtration rate) in control, cecal ligation and puncture (CLP), and CLP + green propolis (CLP + GP) group rats 24 h after the CLP or sham procedure. *p < 0.05 versus CLP + GP; ***p < 0.001 versus control.

The mean GFR (as determined by measuring inulin clearance) was lower in the CLP group than in the control group (0.233 ± 0.033 vs. 0.827 ± 0.054 ml/min/100 g BW; *p* < 0.001). The mean GFR in the CLP + GP group (0.684 ± 0.137 ml/min/100 g BW) was higher than that observed for the CLP group (*p* < 0.05), although it did not differ significantly between the control and CLP + GP groups (Fig. 3). As can be seen in Table 1, serum urea and creatinine were both higher in the CLP group than in the CLP + GP and control groups. Those data correlate well with the inulin clearance results, demonstrating that renal function was restored in the CLP + GP group. As expected, the urinary levels of NGAL were significantly higher in the CLP group than in the control group. Treatment with green propolis had no apparent effect on the urinary levels of NGAL (Table 1).

**Table 1:** Biochemical parameters and Na-K-2Cl cotransporter protein expression.

Parameter	Groups		
	Control	CLP	CLP + GP
Urea (mg/dL)	28 ± 1.7	52 ± 3.7*	37 ± 1.2*^,†^
Creatinine (mg/dL)	0.45 ± 0.05	1.22 ± 0.11*	0.62 ± 0.02^†^
NGAL (ng/mg_creat._)	0.24 ± 0.05	1.43 ± 0.27*	1.38 ± 0.22*
UNa (mEq/mL)	0.51 ± 0.22	1.05 ± 0.51*	0.64 ± 0.53^†^
UK (mEq/mL)	0.88 ± 0.17	1.47 ± 0.52*	1.13 ± 0.22^†^
OSM_U_ (mmoL/kg H_2_O)	695.4 ± 215.8	487.8 ± 204.4*	873.3 ± 430.7^†^
NKCC2 (%)	113 ± 23	47.22 ± 31*	83 ± 27.41^†^

*CLP* cecal ligation and puncture, *GP* green propolis, *NGAL* neutrophil gelatinase-associated lipocalin, *UNa* urinary excretion of sodium, *UK* urinary excretion of potassium, *OSM*_*U*_, urine osmolality, *NKCC2* Na-K-2Cl cotransporter.

*p < 0.05 versus control; ^†^*p* < 0.05 versus CLP.

### Treatment with green propolis completely restored renal tubular function in rats with sepsis

As shown in Table 1, 24-h urinary sodium excretion, 24-h urinary potassium excretion, urine osmolality, and NKCC2 protein expression were all higher in the CLP group than in the CLP + GP and control groups (*p* < 0.05 for all). However, none of those parameters differed significantly between the CLP + GP group and the control group.

### Treatment with green propolis decreased oxidative stress in rats with sepsis

**Figure 4:**
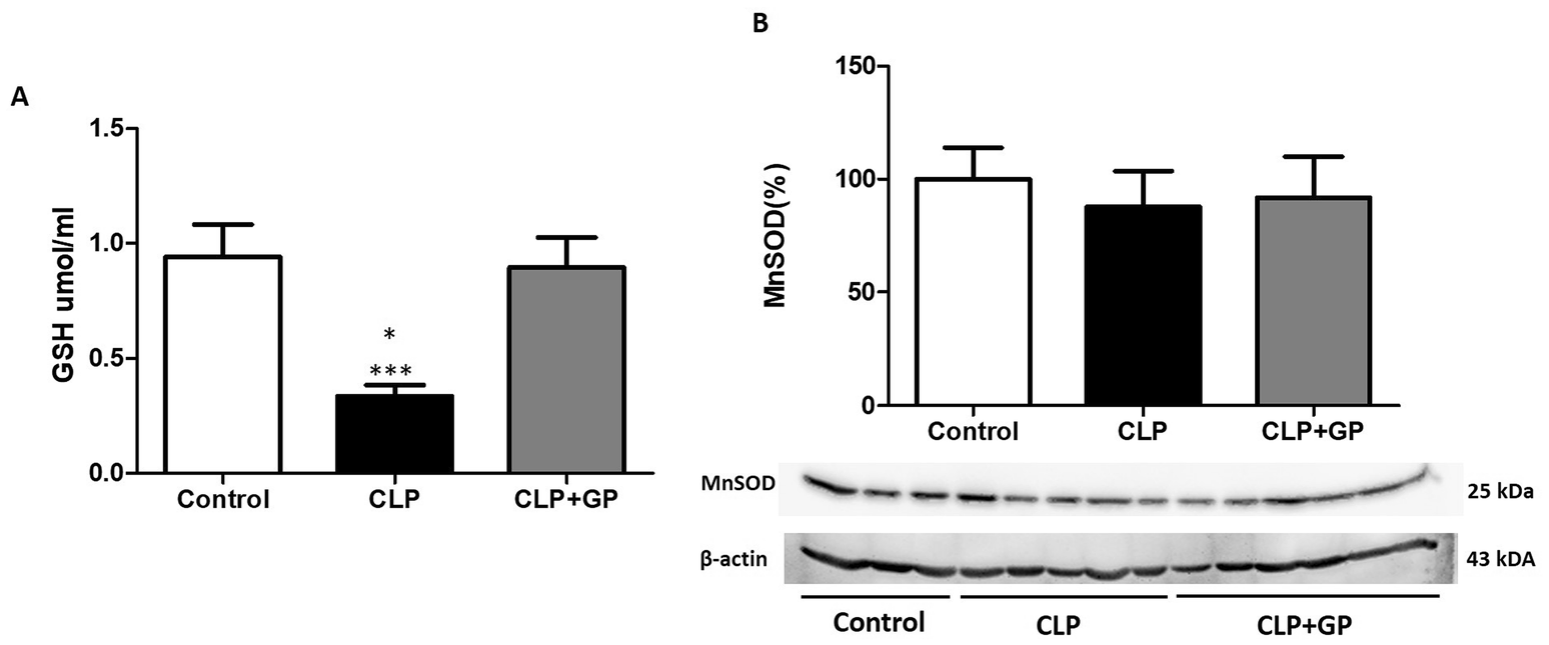
Changes in oxidative stress markers in control, cecal ligation and puncture (CLP), and CLP + green propolis (CLP + GP) group rats 24 h after the CLP or sham procedure. (**A**) Whole-blood reduced glutathione (GSH) levels; (**B**) Immunoblotting of protein expression of manganese superoxide dismutase (MnSOD) in renal tissues; *p < 0.05 versus control; ***p < 0.001 versus CLP + GP.

Mean whole-blood GSH levels were higher in the control group than in the CLP group (0.942 ± 0.139 vs. 0.336 ± 0.148 µmol/ml; *p* < 0.001). The CLP + GP group also presented higher levels of GSH than did the CLP group (0.894 ± 0.131 vs. 0.336 ± 0.148 µmol/ml; *p* < 0.001; Fig. 4A). Expression of MnSOD in renal tissue was slightly higher in the CLP + GP group than in the CLP and control groups (126 ± 18% vs. 112 ± 18% and 100 ± 18.2%, respectively; Fig. 4B).

### Treatment with green propolis decreased upregulation of the TLR4/NF-κB signaling pathway in sepsis-induced AKI

**Figure 5:**
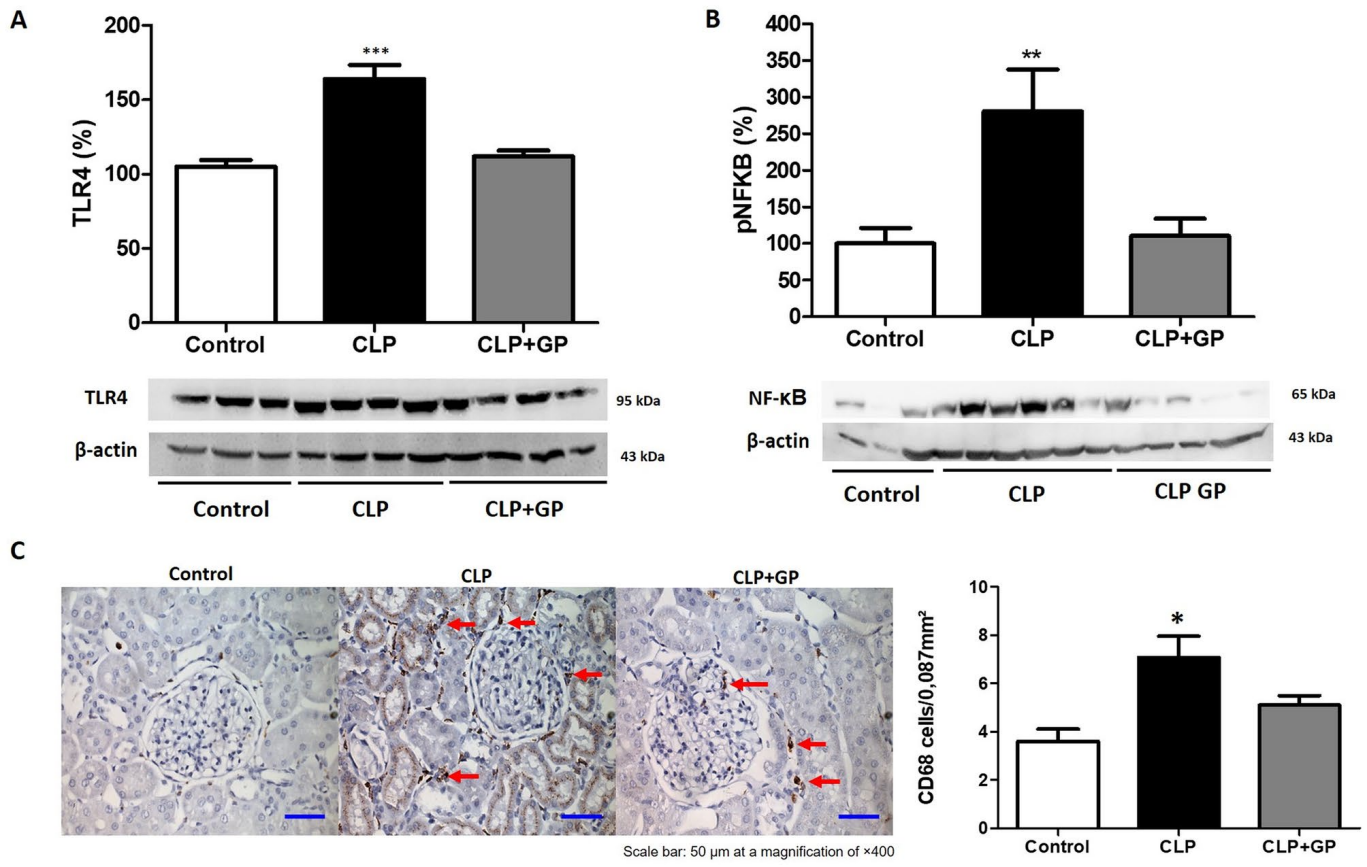
Toll-like receptor 4/nuclear factor-kappa B (TLR4/NF-κB) signaling pathway in renal tissue in control, cecal ligation and puncture (CLP), and CLP + green propolis (CLP + GP) group rats 24 h after the CLP or sham procedure. (**A**) Immunoblotting of TLR4 protein expression in renal tissue; (**B**) Immunoblotting of NF-κB protein expression in renal tissues; (**C**) Macrophage infiltration in renal tissues analyzed by immunohistochemistry. We analyzed 30 grid fields (0.087 mm^2^ each) per kidney cortex, counting the number of infiltrating CD68 + cells in each field, on the basis of which we calculated the mean numbers in the renal cortical tubulointerstitium and the mean counts per kidney. *p < 0.05 versus control and CLP + GP; ***p < 0.001 versus control and CLP + GP; **p < 0.01 versus control and CLP + GP.

The mean renal expression of TLR4 was higher in the CLP group than in the control group (164.0 ± 9.42% vs. 105.0 ± 4.47%; *p* < 0.001), whereas it was lower in the CLP + GP group (111.9 ± 3.97%; *p* < 0.001 vs. the CLP group; Fig. 5A). Similarly, mean NF-κB expression in renal tissue was higher in the CLP group than in the control group (281 ± 57% vs. 100.0 ± 21.0%; *p* < 0.01), whereas it was lower in the CLP + GP group (110.0 ± 24%; *p* < 0.001 vs. the CLP group; Fig. 5B). As can be seen in Table 2, the levels of interleukins in renal tissue were lower in the propolis-treated rats than in the untreated rats.

**Table 2:** Renal tissue levels of cytokines.

Parameter	Groups		
	Control	CLP	CLP + GP
IL-17a (pg/mg)	1.64 ± 0.62	8.56 ± 1.55*	3.37 ± 0.80*^,†^
IL-12p70 (pg/mg)	6.35 ± 2.59	36.27 ± 6.52*	17.01 ± 5.69*^,†^
IL-1β (pg/mg)	7.85 ± 1.02	27.35 ± 7.46*	11.30 ± 4.34*^,†^
TNF-α (pg/mg)	0.67 ± 0.15	2.40 ± 0.52*	0.56 ± 0.13*^,†^
IFN-γ (pg/mg)	2.62 ± 0.50	292.3 ± 84.14*	146.3 ± 34.16

*p < 0.05 versus control; ^†^*p* < 0.05 versus CLP.

### Treatment with green propolis decreased macrophage infiltration into renal tissue

Macrophage infiltration into the renal interstitium, expressed as the mean number of CD68 + cells, was higher in the CLP group than in the control group (7.07 ± 0.88 vs. 3.60 ± 0.50 cells/mm^2^; *p* < 0.05). In addition, the mean number of CD68 + cells in renal tissue in the CLP + GP group (5.10 ± 0.39 cells/mm^2^) was lower than that observed for the CLP group (*p* < 0.05; Fig. 5C).

### Treatment with green propolis decreased apoptosis in renal tissue

**Figure 6:**
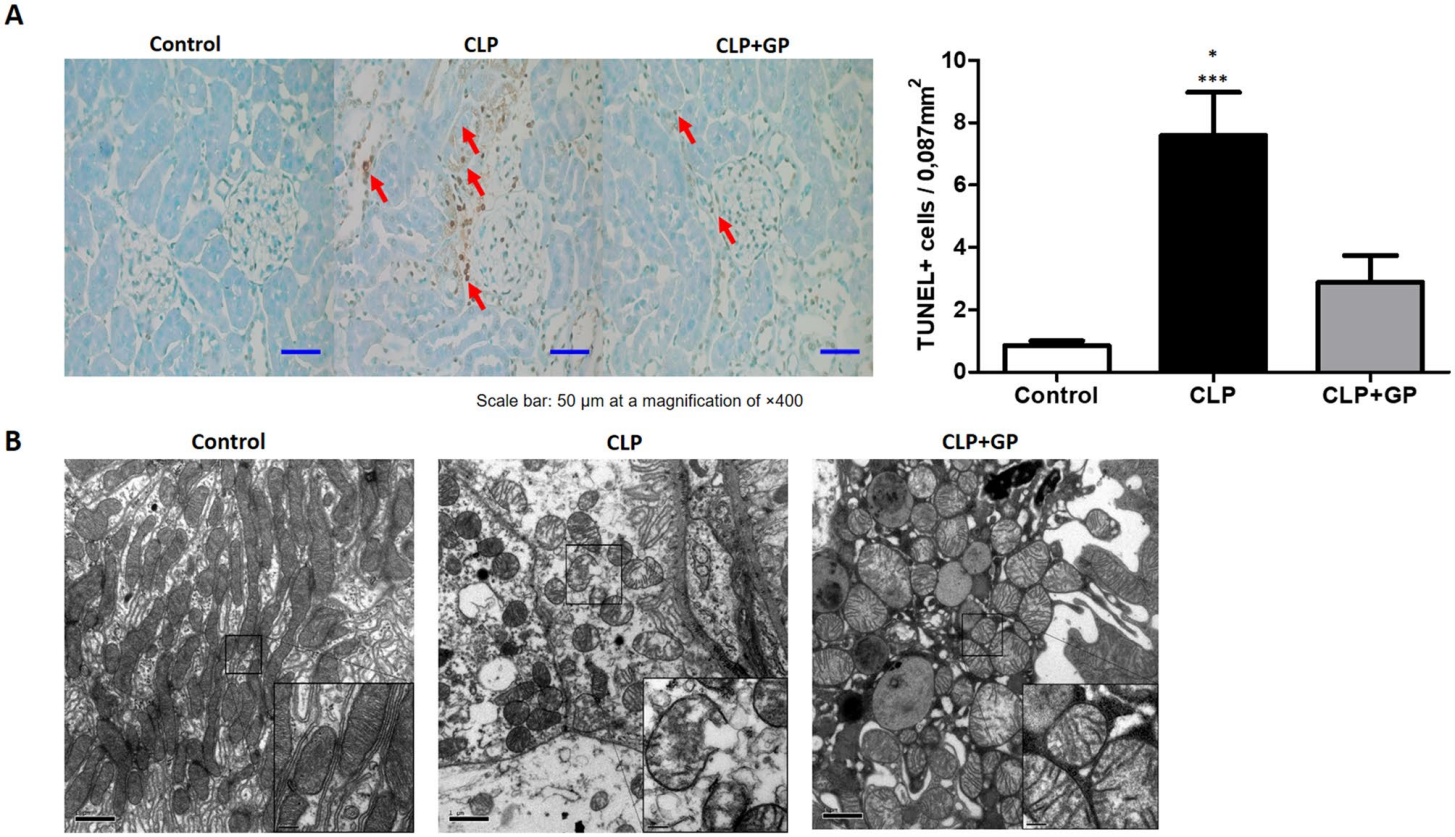
Effects of green propolis on apoptosis and mitochondrial morphology in renal tissues in control, cecal ligation and puncture (CLP), and CLP + green propolis (CLP + GP) group rats 24 h after the CLP or sham procedure. (**A**) Terminal deoxynucleotidyl transferase-mediated deoxyuridine triphosphate nick-end labeling (TUNEL) + cells. We quantified TUNEL + cells in 30 randomly selected fields; (**B**) Mitochondrial morphology. *p < 0.05 versus CLP + GP; ***p < 0.001 versus control.

The mean number of TUNEL + cells was higher in the CLP group than in the CLP + GP and control groups (7.60 ± 1.37 vs. 2.88 ± 0.85 and 0.85 ± 0.15 cells/mm^2^, respectively; *p* < 0.05 for both; Fig. 6A).

### Effects of green propolis on the morphology of renal mitochondria

Absence of the double-membrane mitochondrial system was more common in the CLP group than in the CLP + GP group, as were distorted cristae. Similarly, mitochondrial swelling was most pronounced in the CLP group (Fig. 6B).

### Treatment with green propolis decreased myeloperoxidase activity in the lungs

The mean myeloperoxidase activity in lung tissue was higher in the CLP group than in the CLP + GP and control groups (3.82 ± 0.91 vs. 2.32 ± 1.18 and 0.85 ± 0.63 U/g, respectively; *p* < 0.05 and *p* < 0.001, respectively). In addition, mean myeloperoxidase activity was lower in the control group than in the CLP + GP group (*p* < 0.05).

### Treatment with green propolis decreased macrophage infiltration into lung tissue

**Figure 7:**
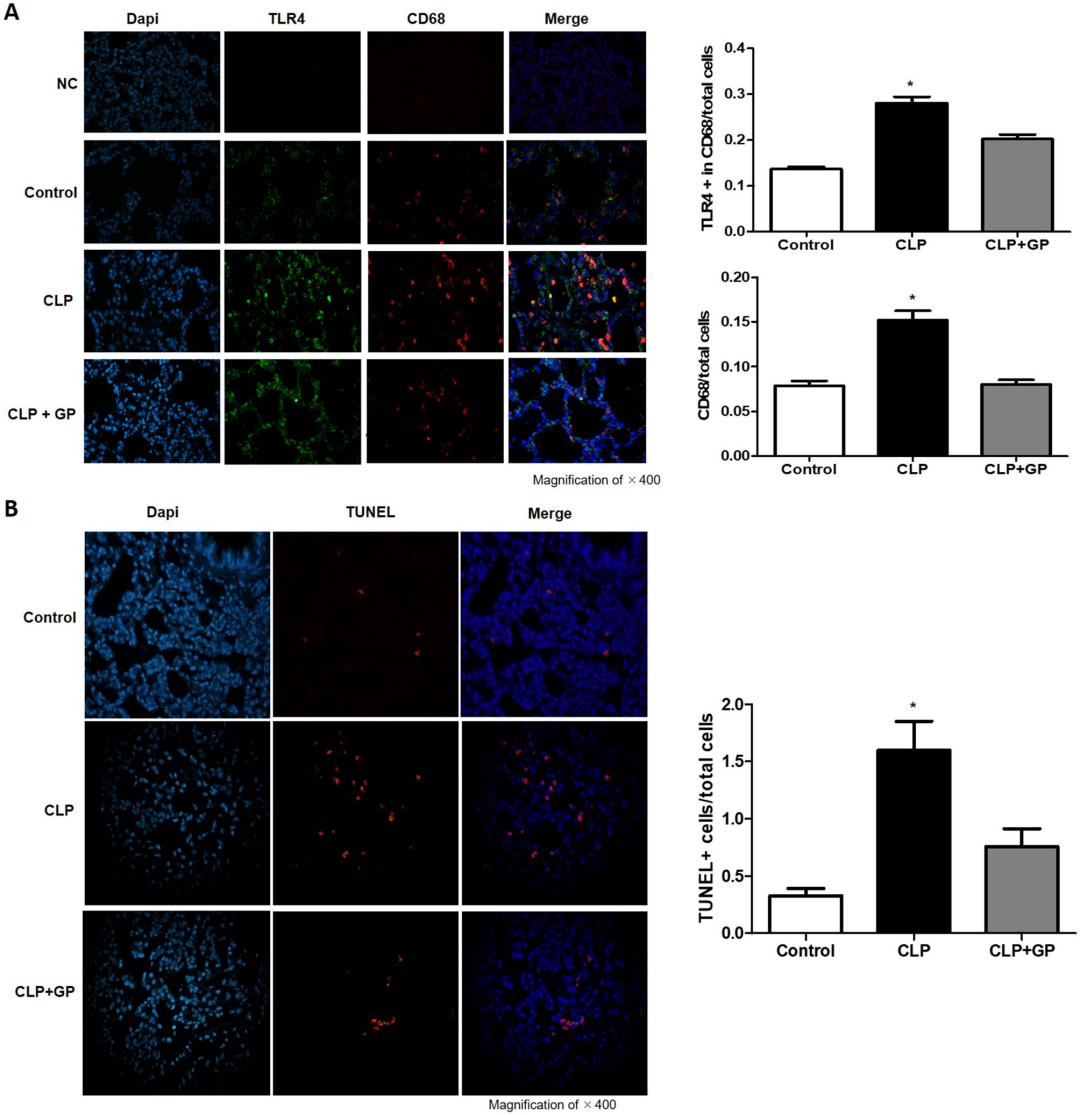
Effects of green propolis on Toll-like receptor 4 (TLR4) protein expression, macrophage infiltration, and apoptosis in lung tissue in control, cecal ligation and puncture (CLP), and CLP + green propolis (CLP + GP) group rats 24 h after the CLP or sham procedure. (**A**) TLR4 expression in macrophage cells. A double-labeling technique was used for CD68 and TLR4 analyzed by immunofluorescence; (**B**) Terminal deoxynucleotidyl transferase-mediated deoxyuridine triphosphate nick-end labeling (TUNEL) + cells analyzed by immunofluorescence. *p < 0.05 versus control and CLP + GP.

Macrophage infiltration into lung tissue, expressed as the mean number of CD68 + cells, was higher in the CLP group than in the CLP + GP and control groups (0.15 ± 0.08 vs. 0.08 ± 0.04 and 0.08 ± 0.035 cells/mm^2^, respectively; *p* < 0.001 for both), as depicted in Fig. 7A.

### Treatment with green propolis decreased upregulation of TLR4 in lung macrophages

The mean expression of TLR4 in lung tissue, presented as the number of macrophages per field that were simultaneously positive for TLR4 and CD68, was higher in the CLP group than in the CLP + GP and control groups (0.28 ± 0.10 vs. 0.20 ± 0.07 and 0.14 ± 0.02, respectively; *p* < 0.001 for both), although it was still higher in the CLP + GP group than in the control group (*p* < 0.05), as can be seen in Fig. 7A.

### Treatment with green propolis decreased apoptosis in lung tissue

In lung tissue, the mean ratio of TUNEL + cells, in relation to the total number of cells, was higher in the CLP group than in the CLP + GP and control groups (1.60 ± 0.56 vs. 0.75 ± 0.38 and 0.44 ± 0.27, respectively; *p* < 0.001 and *p* < 0.05, respectively), as depicted in Fig. 7B.

## Discussion

Here, we have demonstrated that the therapeutic use of green propolis after the induction of sepsis reduced mortality and protected against the inflammatory response in the kidneys and lungs. That protection might be regulated by decreased expression of the TLR4/NF-κB pathway and consequent attenuation of the inflammatory process. In addition, we have shown that the treatment increased the levels of antioxidant protein expression.

Sepsis is the leading cause of AKI worldwide and is independently associated with increased mortality^1,2,4,29^. Inflammation, oxidative stress, endothelial damage, mitochondrial dysfunction, and apoptosis are all involved in the mechanism of such organ injury^7–9^. Therefore, targeting the exaggerated inflammatory response while alleviating the pulmonary and renal injury, together with antibiotic therapy, could be an effective treatment regime for patients with sepsis-induced AKI.

The green propolis used in the present study is rich in flavonoids and phenolic acids^15,30^. One clinical study showed that the plant-derived oral flavonoid baicalin was renoprotective in children with sepsis^31^. Although there have been studies of the use of certain substances isolated from propolis, evidence suggests that whole propolis, with its full complement of substances, is more efficient, because it could act in a number of ways simultaneously^19^.

The CLP model of sepsis is the most widely used of such models worldwide and is considered the best model to mimic human sepsis^32^. It is a polymicrobial infection that provokes systemic inflammation, with multiple organ damage, including the use of antimicrobial therapy and volume resuscitation^5,33^. In our laboratory, we have considerable experience with this model^5,9,26,28,34^.

The CLP model has been shown to reduce the GFR^35^. In the present study, treatment with green propolis increased the GFR in the rats with sepsis-induced AKI. Endothelial dysfunction in sepsis-induced AKI has been attributed to direct endothelial injury and decreased eNOS expression^36^. In the present study, we found that CLP increased the urinary excretion of sodium and potassium. Semiquantitative immunoblotting showed that, as we expected, renal expression of NKCC2 was decreased in rats submitted to CLP and left untreated, as previously shown in other studies, including that conducted by Rodrigues et al.^5^. It has been shown that the expression of NKCC2 in the renal outer medulla is downregulated in rats with lipopolysaccharide (LPS)-induced sepsis^37^. In the present study, treatment with green propolis increased the levels of NKCC2 protein expression.

Endogenous activators of TLR4 can be released by damaged tissues and necrotic cells^38^. Such cells have been found to stimulate the signal transduction into cells through TLR4 and to activate the transcription factor NF-κB. Ultimately, that can trigger the expression of inflammatory cascade effectors, such as interleukins^39^. Li et al.^40^ demonstrated that CAPE, the main active component of propolis, is a potent, specific inhibitor of NF-κB signaling in several cell types. The authors demonstrated the anti-inflammatory effects that CAPE has on LPS-induced human gingival fibroblasts. Their results indicated that CAPE inhibits the production of LPS-induced IL-6, IL-8, inducible nitric oxide synthase, and cyclooxygenase 2, in a dose-dependent manner. Those authors also found that CAPE suppresses LPS-induced activation of TLR4/myeloid differentiation factor 88 and NF-κB. Bueno-Silva et al.^41^ studied the effect of Brazilian red propolis added to cells of the RAW 264.7 murine macrophage-like cell line after activation with LPS. The authors found that red propolis upregulated CD80 and CD86, whereas it downregulated mannose receptor C type 1, indicating M1 polarization. They also found that it attenuated the production of the pro-inflammatory mediators IL-12, granulocyte–macrophage colony-stimulating factor, IFN-γ, and IL-1β in cell supernatants, as well as significantly reducing the LPS-induced upregulation of transcription factors involved in inflammatory signaling (Pdk1, Pak1, Nfkb1, Mtcp1, Gsk3b, Fos, and Elk1). The same authors also showed that the upstream myeloid differentiation factor 88 adaptor-like, which is involved in TLR2 and TLR4 signaling, was downregulated in LPS-activated macrophages treated with red propolis. Given that red propolis inhibited multiple signaling pathways in macrophages involved in the LPS-activated inflammatory process, their data indicate that red propolis is a food source worthy of investigation for the discovery of new bioactive compounds and has the potential to attenuate exacerbated inflammatory diseases. In the present study, treatment with green propolis was found to decrease TLR4 expression in kidneys and lungs, decreasing the numbers of inflammatory cells (macrophages) and the levels of interleukins. Furthermore, in a rat model of LPS-induced sepsis, Korish and Arafa^42^ demonstrated that CAPE reduces the systemic inflammatory response, as well as alleviating hepatic and neuronal cell damage. In the present study, the green propolis-induced decrease in the inflammatory response after CLP could have been responsible for the decrease in apoptosis in the lungs and kidneys, as well as for protecting the renal mitochondria.

## Conclusion

Our results suggest that Brazilian green propolis protects against AKI and lung injury in sepsis. In a therapeutic dose administered 6 h after the induction of sepsis, green propolis reduced inflammation and decreased oxidative stress, promote protection of endothelial cells and mitochondria. In view of the high lethality of sepsis, the use of Brazilian green propolis could be useful as a coadjuvant therapeutic option, because it acts on various pathways involved in the pathogenesis of AKI and lung injury. Our data open the prospect for the use of green propolis in clinical trials.
